# Collagen III Deficiency Following Injury in Female Murine Tendons Alters Matrix Composition, Structure, Organization and Function

**DOI:** 10.1002/jor.70227

**Published:** 2026-05

**Authors:** J. A. Carlson, W. Yen, S. N. Weiss, S. W. Volk, L. J. Soslowsky

**Affiliations:** 1 McKay Orthopaedic Laboratory, University of Pennsylvania, Philadelphia, Pennsylvania, USA; 2 Department of Bioengineering, University of Pennsylvania, Philadelphia, Pennsylvania, USA; 3 School of Veterinary Medicine, University of Pennsylvania, Philadelphia, Pennsylvania, USA

**Keywords:** extracellular matrix, injury, matrix architecture, mechanical properties, remodeling, scar, tendon, type III collagen

## Abstract

Tendons withstand large forces due to an aligned, dense collagen matrix. However, their low cellularity and relative inability to recruit reparative cells post-injury, coupled with a susceptibility to excessive scarring results in loss of tendon structure and function. Type III collagen (COL3) plays a key role in regulating matrix architecture and limiting scar formation following cutaneous injury; however, its role in tendon remains unclear. We examined the impact of reduced COL3 using an established murine *Col3a1* knockdown model. Uninjured tendons in *Col3a1*^+/−^ mice had a broader distribution of fibrils compared to *Col3a1*^+/+^ mice. Fibrils in injured tendons of *Col3a1*^+/−^ mice were larger than those in *Col3a1*^+/+^ mice at 3-weeks post injury but were smaller than their littermates at 6-weeks. Injured *Col3a1*^+/−^ tendons had enhanced fiber alignment at 1- and 6-weeks post-injury and an increase in ɑSMA^+^ myofibroblasts at 3-weeks post-injury. Differential expression of matrix components, as well as markers of cells, cell-ECM interaction, and inflammation were discovered. Anti-inflammatory macrophages were decreased in *Col3a1*^+/−^ tendons 1-week following injury, with no differences between genotypes later in healing. Pro-inflammatory macrophages remained unchanged between genotypes early in healing, but were increased in *Col3a1*^+/+^ tendons compared to *Col3a1*^+/−^ tendons at 3-weeks post-injury. Finally, altered quasistatic mechanical properties was noted in COL3-deficient injured tendons. Our data suggests COL3 plays a complex role in regulating cell phenotype, activity, and fate, as well as collagen matrix architecture in the tendon injury microenvironment, which impacts tendon structure-function post-injury.

## 1 | Introduction

Type I collagen (COL1) is the primary collagen of healthy tendon, while type III collagen (COL3) is a minor constituent that increases following injury [1, 2]. Innate low vascularity and cellularity, as well as aberrant matrix production and architecture, contribute to poor healing and the development of scar tissue with inferior biomechanical properties. While the mechanisms behind persistent scar formation in tendon healing are unknown, we hypothesize that ineffective remodeling of the provisional COL3-rich matrix into an aligned COL1-rich matrix in injured tendons contributes to an abundance of disorganized collagen that comprise the fibrotic scar.

Similar to its role in homeostasis and healing of other collagen-rich tissues (e.g., skin [3, 4], cartilage [5], meniscus [5], bone [6, 7], and vasculature [3, 8]), COL3 is induced following tendon injury [7], suggesting a crucial role early in tendon healing. The loss of the COL1-rich uninjured matrix and filling of the wound void with a COL3-rich provisional matrix is thought to provide a bioactive scaffolding for the recruitment of vulnerary cells that participate in future healing. In skin wounds [9], COL3 supports cellular infiltration to promote early healing, and in later stages, COL3 limits scar formation by suppressing profibrotic myofibroblast activation and by directing an open weave matrix architecture [10]. While COL3-dependent ECM organization and stiffness influence cell behavior in other tissues [4–6, 11], the impact of COL3 expression on fibroblast activation has not been studied in tendon.

Following injury of both skin and tendon, macrophages and mesenchymal cells are recruited, proliferate, and elaborate the post-injury matrix [5, 12]. These cell types modulate the dynamic reciprocity between the cells and their matrix that influence poor versus improved healing. Our previous work revealed that *Col3a1*^+/−^ mice exhibit impairment in granulation tissue formation at 7 days post-cutaneous injury, indicating the importance of COL3 in both cellular recruitment and early matrix deposition [9]. Subsequently, an increase in activation and persistence of myofibroblasts in the COL3-deficient healing niche promotes collagen deposition and remodeling, resulting in highly aligned scars that do not resemble the basket-weave architecture of unwounded skin. While healing of skin and tendon both require recruitment of reparative cells in the early post-injury niche to optimize healing, ideal collagen remodeling to a highly aligned collagen matrix in tendon repair diverges from optimal healing of skin wounds, supporting the innovative concept that COL3 plays unique, temporal roles during tendon healing. An accumulation of COL3 in the post-injury site of tendons has been implicated in poor tendon repair outcomes; however, its overall role in tendon repair is not defined.

We propose that COL3 alters the biophysical and biomechanical features of the collagen fibrous network to optimize the efficiency and quality of tendon repair. In this study, we defined the role of COL3 in directing fibroblast activation during tendon healing and examined how improvements in collagen alignment led to increased mechanical properties. Evaluating the role of COL3 in regulating immune cell and activated fibroblast populations provides a previously unrecognized mechanism by which the COL3 may direct stromal cell activity in the healing tendon. Our study allows us to relate COL3-dependent cellular activities to functional properties and remodeling capability of the tendon throughout healing. The relationship between matrix architecture, composition, and cell-ECM interactions likely shifts over time throughout the different phases of tendon healing. Early in tendon healing, the disorganized matrix supports cellular infiltration and a well-regulated inflammatory phase, that is dampened with a reduction of COL3 in the early provisional matrix [13]. Following this early phase, COL3 inhibits proper cell-ECM interactions, resulting in poor remodeling and alignment, as well as fibrosis progression.

The overall objective of this study was to define the critical role of COL3 in modulating matrix deposition, structure and organization, as well as gene expression and cellular recruitment throughout tendon healing, and resultant effects on matrix function.

## 2 | Methods

### 2.1 | Animal Model and Study Design

The Institutional Animal Care and Use Committee at the University of Pennsylvania approved use and care of the animals used. Female wild-type (*Col3a1*^+/+^) BALB/c and heterozygous *Col3a1*^+/−^ littermates at 90 days of age (*n* = 134) were used. Uninjured (*n* = 46) and injured (bilateral patellar tendon surgery, *n* = 88) groups were randomly assigned. Animals at 1-, 3-, or 6 weeks post-injury, and uninjured age-matched animals were euthanized using carbon dioxide, consistent with current American Veterinary Medical Association Guidelines [14]. Left limbs were utilized for mechanical testing (*n* = 12) and right limbs for histology, gene expression, or transmission electron microscopy (*n* = 4). Both limbs of uninjured mice were assigned to different strain levels for atomic force microscopy (AFM).

### 2.2 | Surgical Induction of Patellar Tendon Injury

Our bilateral patellar tendon (PT) injury model (details published previously) [15, 16] was used. Briefly, the patellar tendon was exposed through a medial incision and isolated with longitudinal incisions following the medial and lateral edge of the tendon. A rubber coated backing was placed behind the tendon, and a 0.75 mm biopsy punch was used to create a full-thickness, partial width defect (~60%) in the midsubstance of the patellar tendon. Mice were administered Buprenorphine SR (0.1 mg/kg) prior to surgery, recovered under a heat lamp and returned to cage activity following skin closure.

### 2.3 | Gene Expression

To assess *Col3a1* levels in *Col3a1*^+/+^ and *Col3a1*^+/−^ mice, as well as to define changes in gene expression induced by COL3 loss during healing, a microfluidic qPCR array (Fluidigm 96.96 Dynamic IFCs) was used [17]. Patellar tendons were excised and stored in RNAlater (Thermo Scientific) at 4°C for 24 h and then transferred to −20°C until sample processing [18]. The method allowed quantification of 96 genes from 40 independent samples (9,216 C_T_ reactions). We analyzed gene expression in uninjured and injured tendons at 1-, 3-, and 6-weeks post-injury within multiple categories listed in Table 1.

For heatmap analysis, ΔCt was calculated by subtracting the gene Ct from the average housekeeper value (*Abl1 and Rps17*) Ct and depicted as a change post-injury from uninjured levels of *Col3a1*^+/+^ tendons. To calculate *Col3a1* fold change, the ΔCt values for the *Col3a1*^+/+^ uninjured group were averaged, and this average was subtracted from each ΔCt value across genotypes and injury time point (ΔΔCt). Finally, fold change was calculated as: fold change = 2^−ΔΔCt^. Tendons (*n* = 3–4) were assessed per genotype and time point. Significant differences in fold change are noted on the heatmap.

### 2.4 | Collagen Localization, Cell Infiltration and Phenotype

#### 2.4.1 | Histology

Knee joints were harvested and fixed for 24 h in 4% PFA, then switched to 30% sucrose for 24 h. Joints and surrounding tissues were embedded in OCT and submerged in methyl butane chilled with dry ice. Cryosections (7 μm) were collected on Cryofilm 2 C (Section-Lab, Japan) and then glued onto slides using chitosan.

#### 2.4.2 | Immunofluorescence

Immunofluorescence (IF) protocols were optimized for targets, including collagen III (COL3; Southern Biotech: 1330-01), α-smooth muscle actin (α-SMA; myofibroblast marker, Sigma-Aldrich: C6198), F4/80 (general macrophage marker, ThermoScientific: MA1-91124), iNOS (M1 pro-inflammatory macrophage marker, Abcam: ab15323) and CD206 (M2 anti-inflammatory macrophage marker, ThermoFisher: PAS-46994). General, M1 and M2 macrophage markers were selected to evaluate the effect of COL3 presence in the injury region on macrophage recruitment and polarization. Briefly, antigen retrieval methods included pepsin (COL3, α-SMA) or proteinase K (iNOS, CD206, F480), followed by host serum blocking and primary antibody incubation overnight at 4°C, with immunofluorescence protocols adapted from those described [11]. On day two, sections were incubated with fluorescently conjugated secondary antibodies for 1 h and coverslips were applied with a mixture of glycerol and PBS including Hoechst nuclear stain diluted to 1:500 in glycerol (ThermoFisher: 33342). Macrophage markers and nuclei were imaged using a Zeiss Axioscan.Z1 with a 20X objective, with appropriate excitation wavelengths and exposure times. A region of interest (ROI) was defined by the boundary of the injured tissue on each slide and Integrated Density was calculated using the IntDen Function in ImageJ on the outlined area. Integrated density was normalized per area for group comparisons as the injury size differed between groups. Multiplexed staining protocol for COL3 and α-SMA was followed with multiphoton imaging.

#### 2.4.3 | Multiphoton and Second Harmonic Generation (SHG) Imaging and Analysis

An ROI was defined by the boundary of the injured tissue on each slide. COL3 and α-SMA were imaged on an upright Leica SP8 confocal/multiphoton microscope (Leica Microsystems, Mannheim, Germany). α-SMA fluorescence was captured with a 555 nm wavelength laser and COL3 with a 647 nm wavelength laser (PMT detectors). Imaging of fibrillar collagen was performed by tuning the Coherent Chameleon Ultra II Ti:Sapphire laser (Coherent Inc., Santa Clara, CA) to 910 nm and collecting both forward and backward SHG signal on PMT-RLD and HYD-RLD detectors. Z-stacks were taken at 1 μm intervals to capture the depth of the tendon in uninjured mid-substance or through the injury site. Integrated density was calculated using ImageJ, as explained above.

For uninjured sections, a representative ROI (0.15 mm × 0.3 mm) was drawn around an uninterrupted area of uninjured tissue. Collagen fiber alignment was calculated using Fast Fourier transform (FFT). An ellipse was fit around the processed FFT-power plots and the FFT Aspect Ratio was calculated by dividing the major axis by the minor axis. Fiber orientation angles (within an image) were analyzed with OrientationJ (ImageJ plug-in) [11, 19]. Images were first transformed to 8-bit and then ROIs were selected for analysis. Distributions of fiber orientation were analyzed. The number of fibers within ±15° of the modal angle per group was used for statistical analysis.

Lastly, CT Fire [20] (https://loci.wisc.edu/ctfire/) was utilized to analyze width, length, and straightness of collagen fibers within the injury sites (*n* = 4 tendons per genotype and time point). After denoting individual ROIs surrounding the injury site, the program extracts individual collagen fibers and measures structural properties.

### 2.5 | Matrix Structural Analyses

#### 2.5.1 | Tendon Fibril Structure

Tendons were analyzed using transmission electron microscopy and a camera (TEM, using a JEOL 1400, JEOL Ltd., Tokyo, Japan) as described [16, 18]. Patellar tendons from 90 day-old mice (*n* = 4 mice/genotype/time point) were harvested, underwent fixation, dehydration, cut in half through the injury site, and embedded. Ultra-thin cross sections were obtained and 15 images per sample were collected at 60,000X magnification, with an average of 500 fibrils assessed per image (*n* = 4 per group). Fibril structure was defined as described [18].

### 2.6 | Matrix Functional Analyses

#### 2.6.1 | Biomechanical Testing

##### Sample preparation.

Patella-PT-tibia complexes were dissected to isolate the patella tendon from surrounding tissue. Measurement of cross-sectional area used a custom laser device [13]. Tendons (*n* = 8–11) were assessed per genotype and time point. Tendons were stamped into a dog-boned shape and mounted with the bone at a physiologically relevant angle of 35° angle as described [18].

##### Viscoelastic testing.

Specimens were tested on an Instron 5848 utilizing the following protocol [1]: 1) preload to 0.05 N, 2) 10 cycles of preconditioning to 1.5% strain at 0.25 Hz, 3) stress relaxation to 3% strain at 5%/s, 4) sinusoidal frequency sweep, 5) stress relaxation to 6% strain at 5%/s, 6) sinusoidal frequency sweep, 7) return to gauge length and 8) ramp to failure at 0.05%/s. Each frequency sweep consisted of 10 cycles of 0.125% amplitude sinusoidal strain at frequencies of 0.01, 0.1, 1, 5 and 10 Hz. The dynamic modulus, |E*| (stress amplitude divided by the strain amplitude), and the phase angle, δ (between the stress and strain), were computed at each frequency and strain level using a Matlab program [19–21].

#### 2.6.2 | Fiber Re-Alignment Measurement

Collagen fiber re-alignment was quantified as described [8, 22]. During the ramp to failure test, optical and polarized images were acquired for strain analysis as described [8, 23]. Circular variance (VAR), a measure of the distribution of fiber alignment [8, 23], was measured to determine if realignment occurred during each portion of the mechanical test.

#### 2.6.3 | Collagen Fibril Deformation and Sliding

Fibril deformation and sliding were quantified using AFM and a flash-freeze protocol [20, 24]. Patella-patellar tendon-tibia complexes were dissected, and cross-sectional area was measured using a custom laser device as described [13]. The tibia-PT-patella complex was loaded into an Instron 5543. Gauge length was optically measured for strain calculations [18]. Briefly, tendons were subjected to preconditioning, a 60 s hold, and then ramp to 1% or 10% strain at a rate of 0.1%/s. Tendons were then flash frozen, cut from the grip, and embedded in chilled OCT and submerged in methyl butane chilled with dry ice. Blocks were then sectioned at 20 μm to obtain 5 slides with 3–4 sections per slide. For fibril deformation and sliding, imaging was performed in air by tapping mode imaging using NCHV-A probes (nominal spring constant *k* ≈ 42 N/m, radius *R* ≈ 10 nm) and a Dimension Icon AFM [25]. Tendons (*n* = 5–6/genotype at 1% and 10% strain) were scanned at 4–6 sections throughout the tendon depth within the midsubstance. Custom software was used to measure the d-period length for multiple fibrils in a single image [26].

### 2.7 | Statistics

Two-way ANOVAs with post-hoc Bonferroni tests were used to assess the effects of genotype, injury time point, and their interaction on gene expression, fiber alignment measures, integrated density, and viscoelastic and quasistatic mechanical properties. Two-way repeated measures ANOVAs with post-hoc Bonferroni tests were used to assess the differences in realignment between genotypes over increasing strain levels. Two-way ANOVAs with post-hoc Bonferroni tests were used to assess the effects of genotype (COL3 expression), strain level (1% or 10%), and their interaction on fiber d-period average and variance. Collagen fibril diameter distributions were compared by genotype using Kolmogorov-Smirnov tests. Significance was set at *p* ≤ 0.05.

## 3 | Results

### 3.1 | COL3 Expression and Immunoreactivity are Significantly Decreased at Early Post-Injury Time Points in Tendons of *Col3a1*^+/−^ Mice Compared to *Col3a1*^+/+^ Littermates

**FIGURE 1 |:**
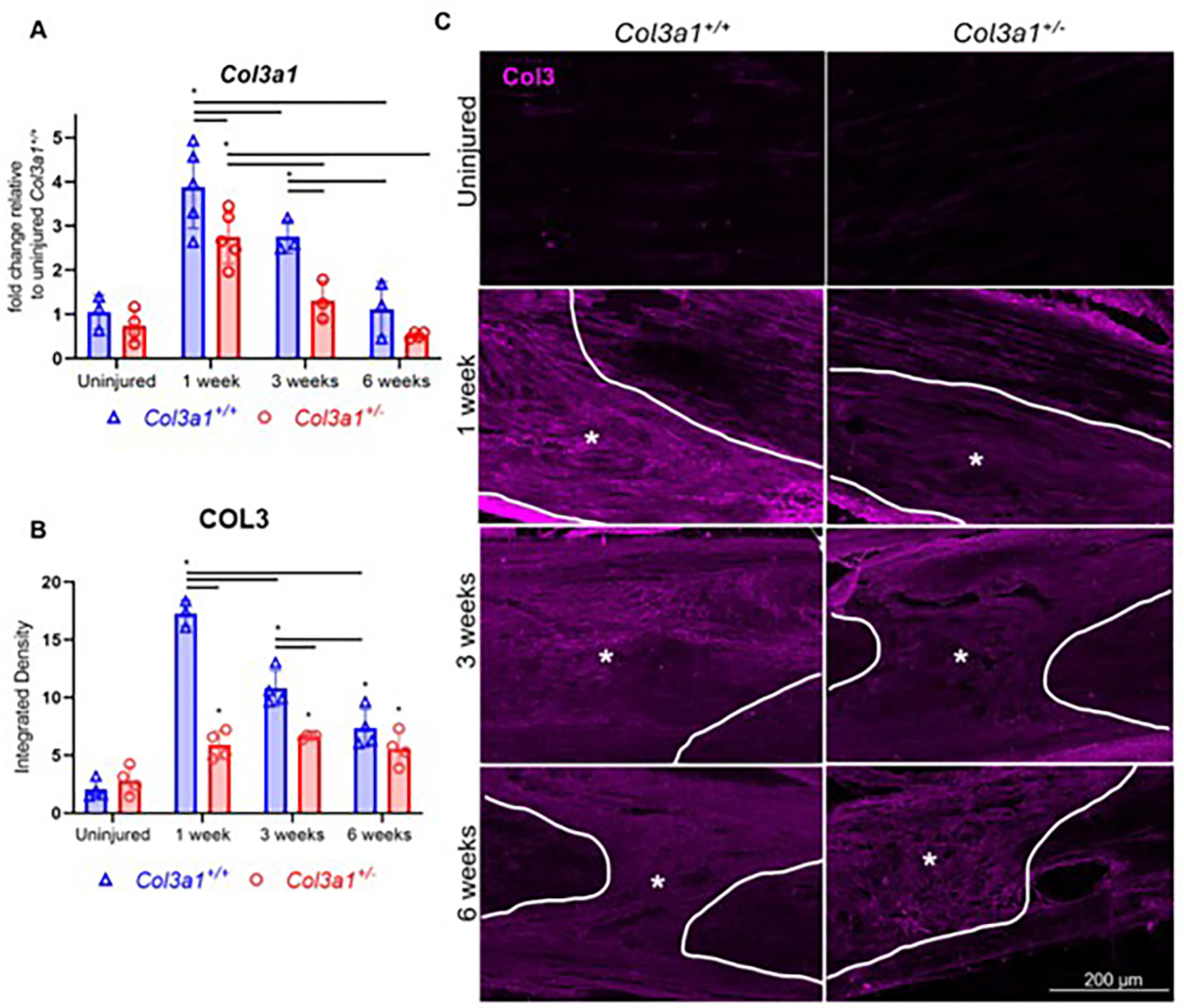
COL3 mRNA expression and immunoreactivity are decreased at early post-injury time points in tendons of *Col3a1*^+/−^ mice compared to *Col3a1*^+/+^ littermates. (A) *Col3a1* expression is increased at 1-week following tendon injury in both genotypes and is decreased in tendons from *Col3a1*^+/−^ compared to *Col3a1*^+/+^ mice at 1- and 3-weeks following injury. (B) COL3 immunoreactivity was decreased in histological sections of *Col3a1*^+/−^ tendons at 1- and 3-weeks post-injury relative to those from *Col3a1*^+/+^ mice. Immunoreactivity was increased following injury in both genotypes when compared to uninjured controls. (C) Representative images of uninjured and injured *Col3a1*^+/+^ and *Col3a1*^+/−^ tendons (white lines outline the injury region that was quantified, as indicated by an *). Solid lines in graphs denote significance of *p* ≤ 0.05. *denotes significant differences between injury group and corresponding uninjured group. *n* = 3–4 samples/genotype/time point.

Given that global knockout of *Col3a1* during development results in perinatal lethality, we utilized an established murine model of COL3 haploinsufficiency to assess the impact of COL3 loss on tendon repair [4–6, 21, 27, 28]. Interestingly, *Col3a1* expression was not different in uninjured tendons of *Col3a1*^+/−^ mice compared to *Col3a1*^+/+^ littermates, likely due to low baseline COL3 levels in uninjured tendons and the small sample size and assay variability. However, *Col3a1* expression was decreased in *Col3a1*^+/−^ tendons compared to *Col3a1*^+/+^ tendons at both 1- and 3-weeks post-injury, highlighting feasibility of this injury model to investigate our hypothesis. As anticipated, *Col3a1* expression increased post-injury in both *Col3a1*^+/+^ and *Col3a1*^+/−^ tendons, however, *Col3a1* expression was increased through 3-weeks post-injury in *Col3a1*^+/+^ tendons, while its expression in *Col3a1*^+/−^ tendons had a dampened response with an earlier return to uninjured levels by 3-weeks post-injury (Figure 1A). To assess changes in COL3 in this model, integrated density of immunolabelled COL3 was measured in uninjured tendons and within the injury region of tendons at 1-, 3-, and 6-weeks post-injury. Consistent with changes in gene expression, COL3 immunoreactivity was similar between genotypes in uninjured tendons and was induced after injury in both genotypes. As anticipated, levels in *Col3a1*^+/−^ tendons were decreased compared to *Col3a1*^+/+^ at 1- and 3-weeks post-injury (Figure 1B,C). Levels remained elevated over pre-injury levels throughout all time points post-injury in both genotypes (Figure 1B,C). These findings support the use of this model to evaluate the requirement and roles of COL3 in tendon healing.

### 3.2 | COL3 Content and Time From Injury Impact Fibril Diameter Distributions in Healing Tendons

**FIGURE 2 |:**
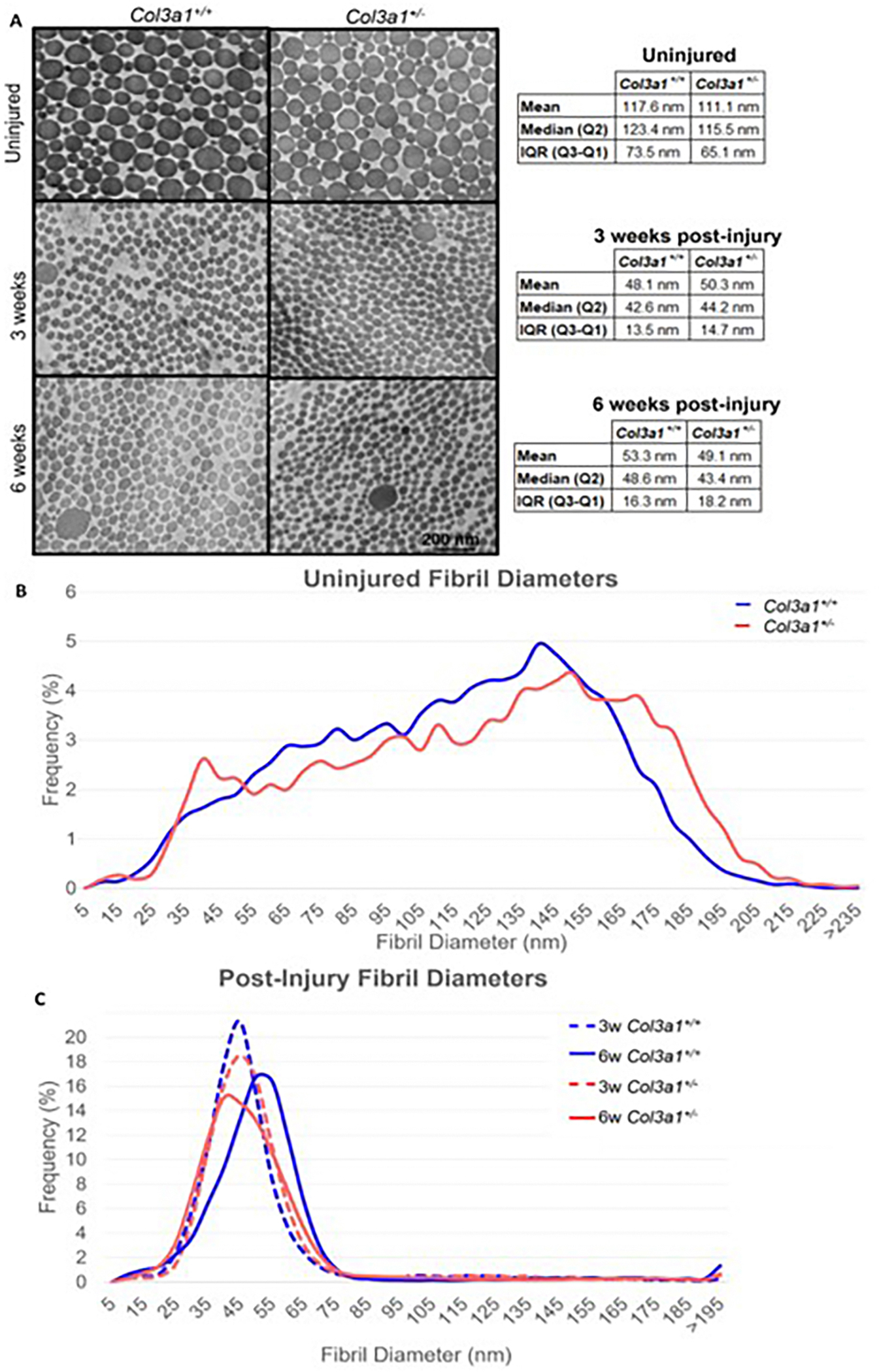
Collagen Fibril Diameter Distributions in uninjured and injured tendons of *Col3a1*^+/+^ and *Col3*^+/−^ mice. (A) Representative TEM images of uninjured and injured *Col3a1*^+/+^ and *Col3a1*^+/−^ tendons in cross-section. A shift to smaller fibrils is seen within each genotype following injury (3- and 6-weeks post injury). (B) Uninjured COL3-deficient (*Col3a1*^+/−^) tendons had an increased population of larger diameter fibrils. (C) Post-injury COL3-deficient tendons had an increased population of larger fibrils at 3-weeks and smaller fibrils at 6-weeks. Distributions were compared by genotype using Kolmogorov-Smirnov tests. Significance was set at *p* ≤ 0.05. 15 images per sample were analyzed, with an average of 500 fibrils assessed per image. *n* = 4 tendons/group.

Due to the influence of COL3 on fibril deposition and lateral growth in skin and the vasculature [3], of SHG-positive collagen we examined the impact of COL3-deficiency on collagen fibril diameter in uninjured and healing tendons over time post-injury (Figure 2). Fibril diameters were measured from TEM images presented in Figure 2A. Uninjured *Col3a1*^+/−^ tendons had a broader fibril diameter distribution than *Col3a1*^+/+^ tendons, with an increased population of larger fibrils (Figure 2B). Post-injury both genotypes showed a dramatic shift to a more uniform population of smaller diameter fibrils. *Col3a1*^+/+^ fibril diameters increased from 3- to 6-weeks post-injury. At 3-weeks post-injury, *Col3a1*^+/−^ tendons had a slightly greater population of larger fibrils compared to *Col3a1*^+/+^ tendons (Figure 2C). Interestingly, at 6-weeks post-injury, *Col3a1*^+/−^ tendons had an increased population of smaller diameter fibrils when compared to *Col3a1*^+/+^ tendons.

### 3.3 | COL3 Loss Promotes Collagen Fiber Alignment in Healing Tendons

**FIGURE 3 |:**
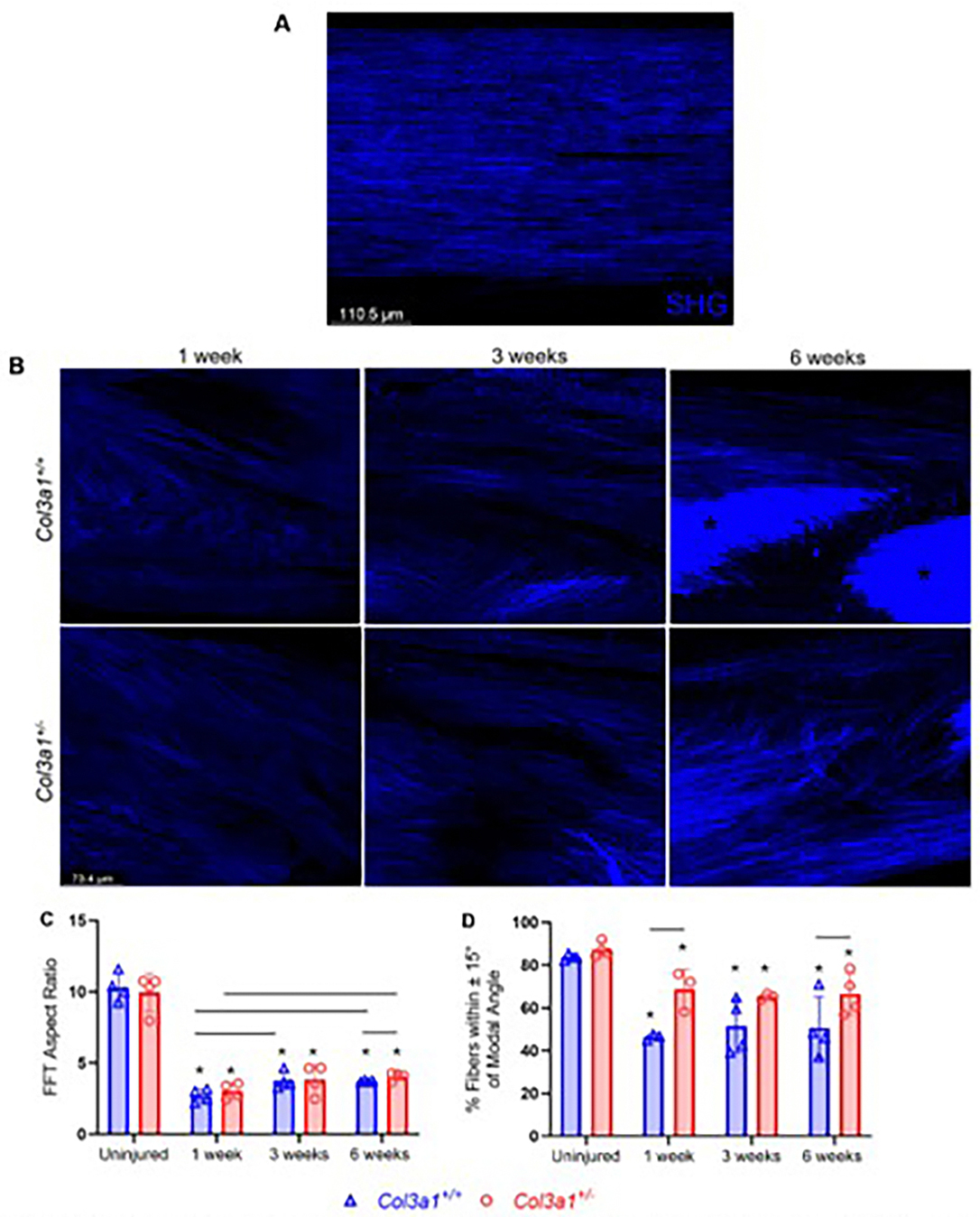
COL3 loss promotes collagen fiber alignment following injury. (A) Representative SHG image of fibrillar collagen in an uninjured wild-type (*Col3a1*^+/+^) tendon. (B) Representative SHG images of injured *Col3a1*^+/+^ and COL3-deficient (*Col3a1*^+/−^) tendons following injury. Entire areas of injury sites were analyzed. *denotes excluded areas of uninjured tendon in the 6-week image of *Col3a1*^+/+^. (C) FFT aspect ratio increased at 6-weeks post-injury in COL3-deficient (*Col3a1*^+/−^*)* tendons compared to *Col3a1*^+/+^. (D) A greater number of fibers were within 15° of the modal angle at 1- and 6-weeks post-injury in *Col3a1*^+/−^ compared to *Col3a1*^+/+^ tendons. Solid lines in graphs denote significance of *p* ≤ 0.05. *denotes differences between injury group and corresponding uninjured group. *n* = 3–4 samples/genotype/time point.

To further understand the impact of COL3-deficiency on collagen fiber architecture, fiber alignment, straightness, length and width were evaluated (Figure 3). Despite enhanced fiber alignment associated with COL3-deficiency and uninjured status compared to injured *Col3a1*^+/+^ tendons, fiber width, length and straightness were not affected (Supplemental Figure 1). Alignment was reduced in injured regions of tendons of both *Col3a1*^+/+^ and *Col3a1*^+/−^ mice at all time points post-injury compared to pre-injury (Figure 3A,B), as assessed by both FFT aspect ratio (Figure 3C) and % fibers within 15° of the modal angle of SHG-positive collagen fibers (Figure 3D). In *Col3a1*^+/+^ tendons, healing promoted alignment from 1- to 3-weeks post-injury. This degree of alignment was maintained between 3- and 6-weeks post-injury in *Col3a1*^+/+^ mice (Figure 3C). COL3-deficiency promoted alignment at 1- and 6-weeks post-injury when compared to *Col3a1*^+/+^(Figure 3D).

### 3.4 | COL3-Deficiency Increases ɑSMA^+^ Integrated Density in Injured Compared to *Col3a1*^+/+^ Tendons in Early Healing

**FIGURE 4 |:**
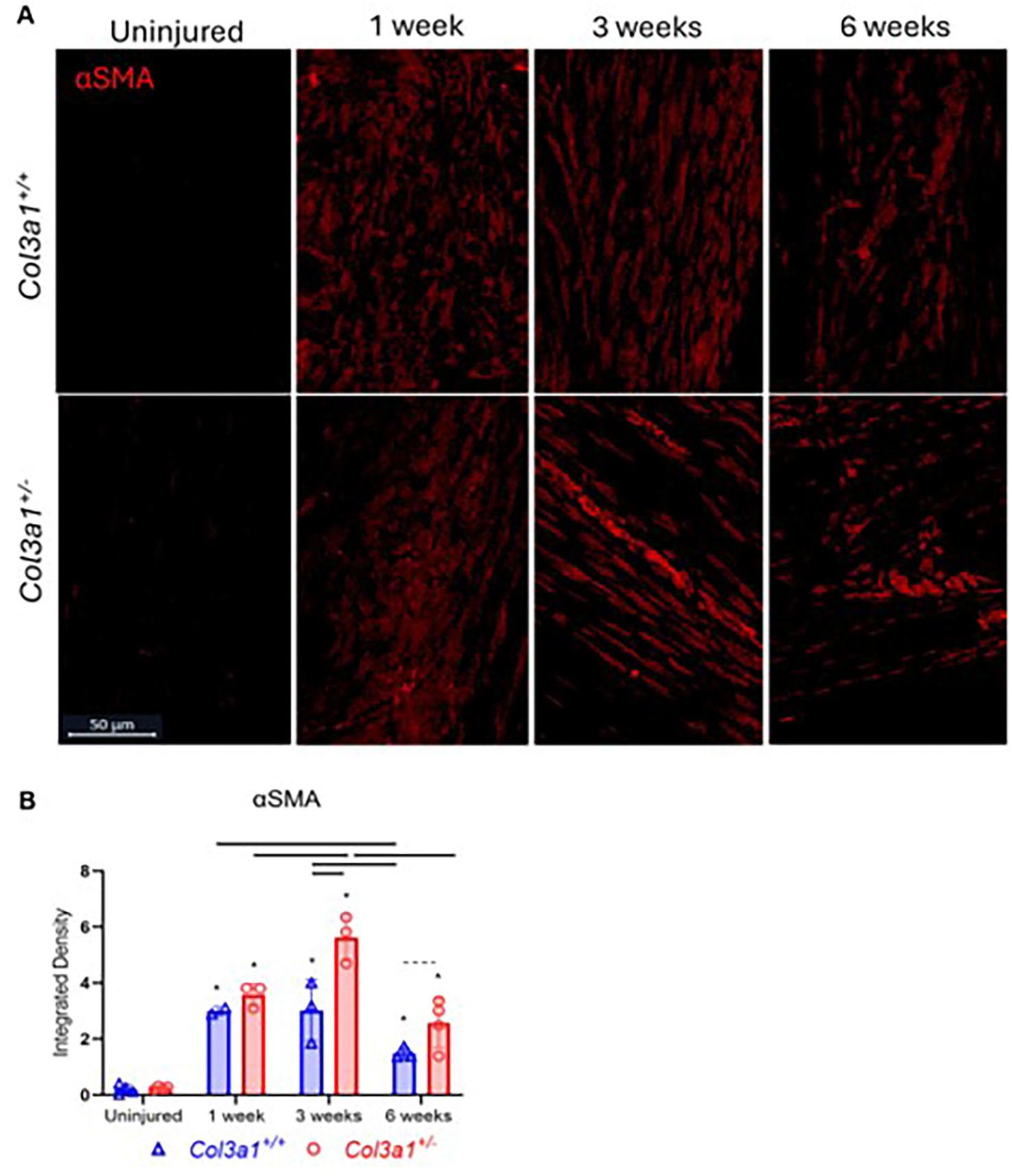
COL3-deficiency increases αSMA^+^ myofibroblasts in healing tendons. (A) Myofibroblast density, as determined by αSMA^+^ immunoreactivity, was increased in *Col3a1*^+/−^ tendons at 3-weeks post-injury compared to *Col3a1*^+/+^ tendons. (B) Representative images of anti-αSMA immunofluorescence in histologic sections of uninjured and injured *Col3a1*^+/+^ and *Col3a1*^+/−^ tendons. *n* = 3–4 samples/genotype/time point.

Given enhanced collagen fiber alignment in both healing tendons and skin associated with COL3-deficiency, and COL3's known role in suppressing myofibroblast activation and persistence in other tissues [9, 11], we examined ɑSMA^+^-myofibroblast density, with representative images of anti-αSMA immunofluorescence shown in Figure 4A and quantification in Figure 4B. No difference in myofibroblast density was seen between genotypes in uninjured tendons, however, as anticipated, an induction of ɑSMA^+^ immunoreactivity was seen in both genotypes at all time points post-injury when compared to uninjured tendons (Figure 4B, denoted by *). Mean myofibroblast integrated density in *Col3a1*^+/+^ tendons increased to ~14.5x relative to uninjured tendons by 1-week post-injury and then decreased by 6-weeks post-injury (Figure 4B). In contrast, myofibroblast density in healing *Col3a1*^+/−^ tendons continued to increase to 3-weeks post-injury, reaching a maximum of ~22x uninjured mean integrated density levels, before decreasing between 3- and 6-weeks post-injury (Figure 4B). Notably, there was an increase in myofibroblast density in healing tendons of *Col3a1*^+/−^ mice compared to *Col3a1*^+/+^ littermates at 3-weeks post-injury.

### 3.5 | Differential Gene Expression Is Associated With COL3-deficiency in Uninjured and Healing Tendons

**FIGURE 5 |:**
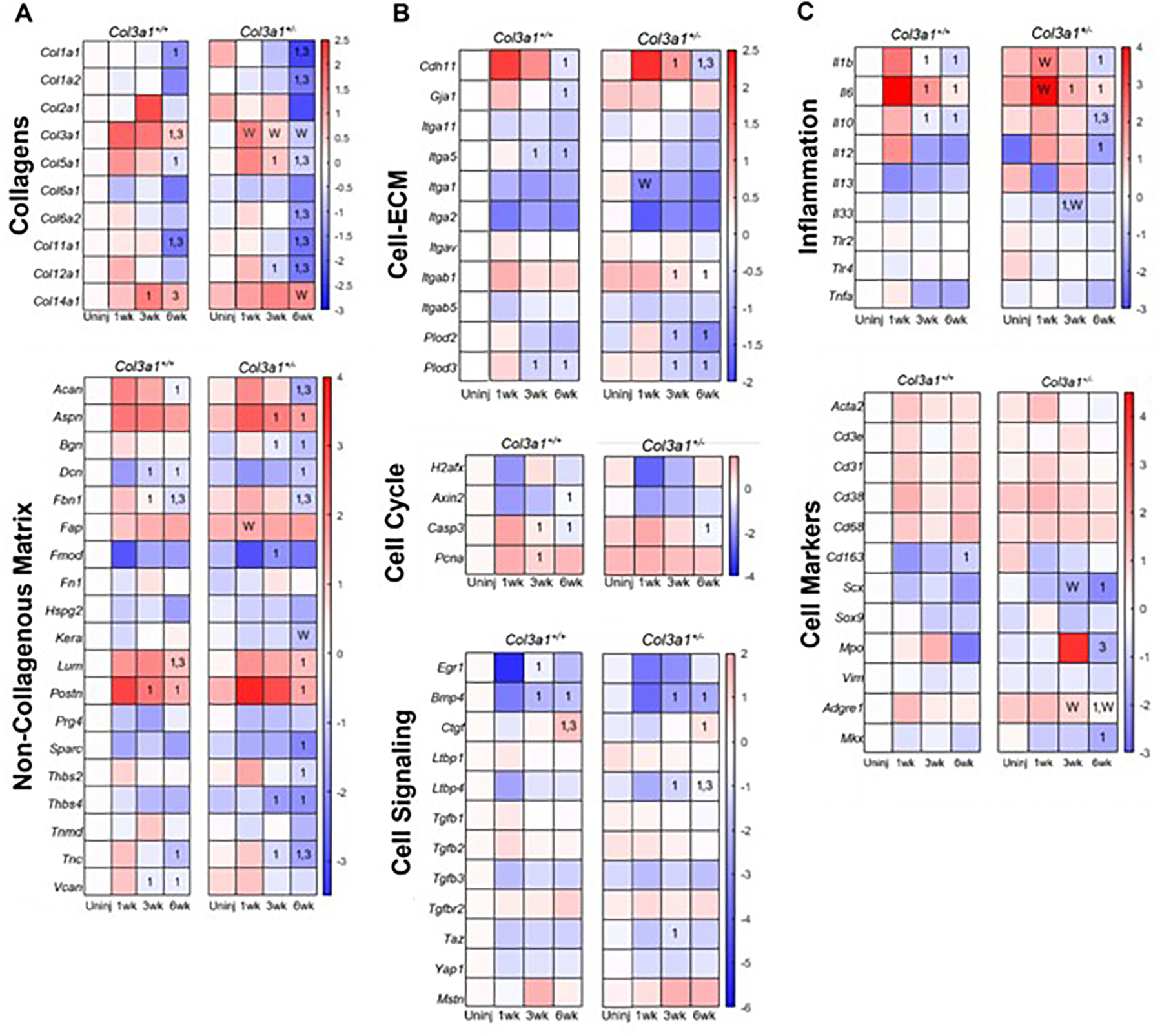
Regulation of gene expression by time from injury and COL3-deficiency in murine tendons. Heatmaps for each category were prepared using *Col3a1*^+/+^ uninjured gene expression as the baseline level. ‘W’ indicates a significant difference from *Col3a1*^+/+^ expression in COL3-deficient tendons at the time point noted. ‘1’ and ‘3’ indicate significant temporal differences in expression within genotype between the time point denoted and expression at 1- and 3-weeks, respectively. Significance is set at *p* ≤ 0.05. *n* = 3–4 samples/genotype/time point. (A) Collagens; Non-collagenous Matrix; (B) Cell-ECM; Cell Cycle; Cell Signaling (C) Inflammation; Cell Phenotype Markers.

We hypothesized that phenotypic changes associated with COL3-deficiency in tendons of *Col3a1*^+/−^ mice would lead to changes in gene expression in both uninjured and injured tendons, compared to those in *Col3a1*^+/+^ mice. *Col3a1* expression was decreased in healing tendons of *Col3a1*^+/−^ mice relative to *Col3a1*^+/+^ mice, as expected (Figure 5A). Comparison of genes associated with non-collagenous matrix revealed few differences except for a relative increase in fibroblast activation protein (*Fap*) at 1-week post-injury and a decrease in keratocan (*Kera*) at 6-weeks post-injury in *Col3a1*^+/−^ tendons compared to *Col3a1*^+/+^ tendons. Markers of cell-ECM interactions followed similar expression patterns (Figure 5B). Cell cycle markers showed alterations across time points in *Col3a1*^+/+^ tendons but minimal changes post-injury in *Col3a1*^+/−^ tendons (Figure 5B). Cell signaling genes were also not differentially expressed in *Col3a1*^+/+^ and COL3-deficient tendons (Figure 5B). Expressions of proinflammatory cytokines, *Il-1β* and *Il-6*, were increased at 1-week post-injury in COL3-deficient tendons compared to *Col3a1*^+/+^ tendons (Figure 5C). *Il-6* expression decreased by 3-weeks post-injury from 1-week levels in both genotypes. *Il-1β* expression decreased by 3-weeks post-injury from 1-week levels in *Col3a1*^+/+^ tendons and by 6-weeks post-injury in COL3-deficient tendons. Pro-fibrotic cytokine, *Il-33*, was downregulated in COL3-deficient tendons at 3-weeks post-injury compared to *Col3a1*^+/+^ tendons and 1-week levels. Lastly, Scleraxis (*Scx*) expression was decreased at 3-weeks in *Col3a1*^+/−^ compared to *Col3a1*^+/+^ tendons (Figure 5C). Adhesion G Protein-Coupled Receptor E1 (*Agre1*), also known as F4/80, was increased at 3-weeks post-injury but decreased 6-weeks post-injury in *Col3a1*^+/−^ relative to *Col3a1*^+/+^ tendons.

### 3.6 | Macrophage Density Is Temporally Altered by COL3-Deficiency in Healing Tendons

**FIGURE 6 |:**
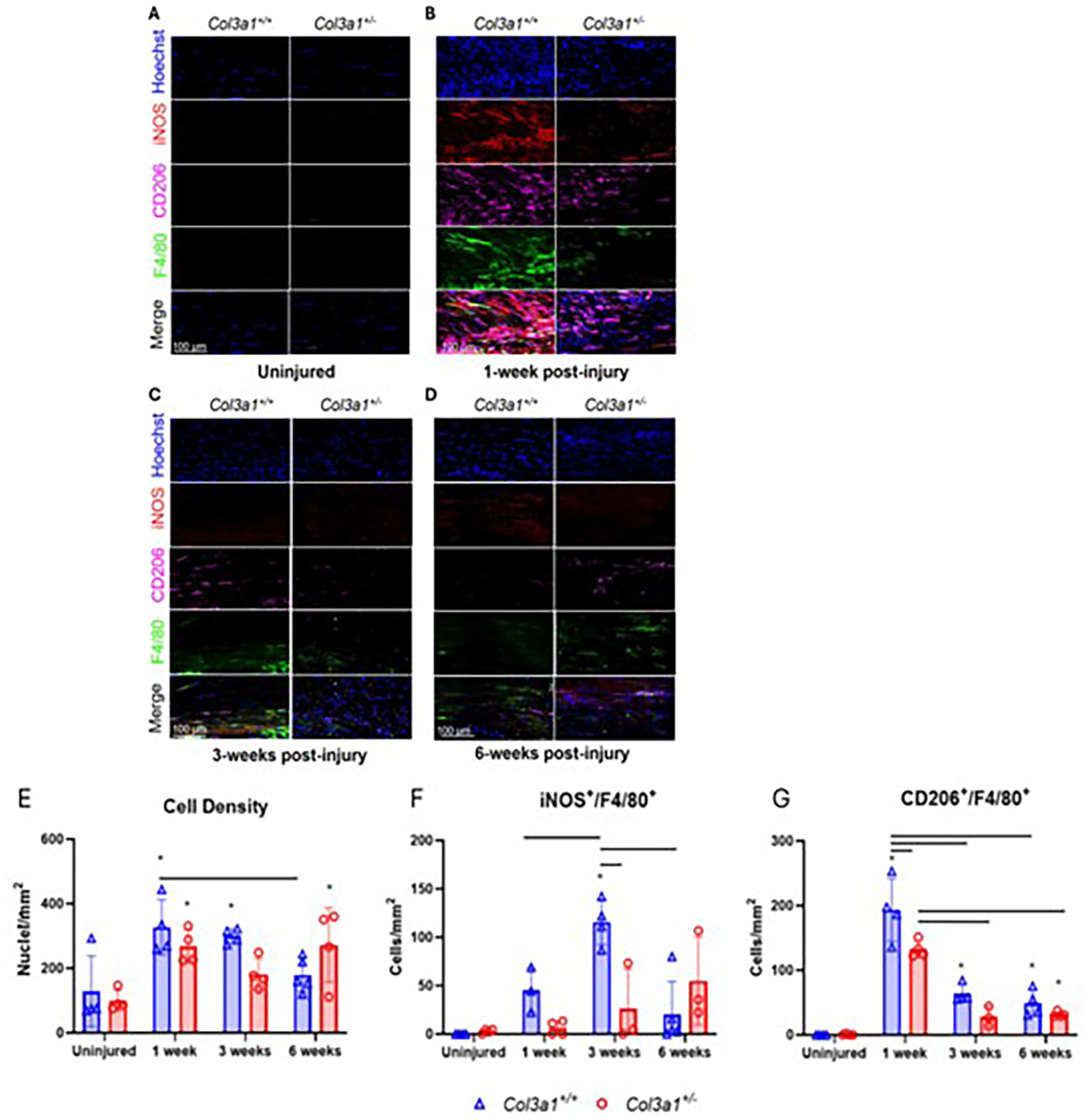
Representative images and quantification of macrophage staining in the injury region or uninjured midsubstance tendon. (A–D) iNOS (red) was evaluated as a marker of a pro-inflammatory phenotype, CD206 (pink) as an anti-inflammatory marker, and F4/80 (green) as a general macrophage marker. (E) Cell density increased post-injury at 1- and 3-weeks post-injury in *Col3a1*^+/+^ tendons, and 1- and 6-weeks post-injury in *Col3a1*^+/−^ tendons. (F) COL3-deficiency resulted in reduced iNOS^+^/F4/80^+^ staining 3-weeks post-injury and reduced CD206^+^/F4/80^+^ staining 1-week post-injury (G). Solid lines denote statistical significance of *p* ≤ 0.05. *Denotes significant increase from corresponding uninjured group. *n* = 3–4 samples/genotype/time point.

Representative images stained for F4/80 (general macrophage marker, green), CD206 (M2 anti-inflammatory macrophage marker, magenta) and iNOS (M1 pro-inflammatory macrophage marker, red), with corresponding integrated density measurements (Figure 6A–D), indicate that cell density increased post-injury at 1- and 3-weeks post-injury in *Col3a1*^+/+^ tendons, and 1- and 6-weeks post-injury in *Col3a1*^+/−^ tendons compared to uninjured tendons. There is a smaller population of CD206^+^/F4/80^+^ cells and iNOS^+^/F4/80^+^ cells within the injury site at 1- and 3-weeks post-injury, respectively, in *Col3a1*^+/−^ compared to *Col3a1*^+/+^ tendons (Figure 6F,G).

### 3.7 | COL3-Deficient Tendons Had Increased Quasistatic Mechanical Properties Compared to *Col3a1*^+/+^ Tendons Throughout Healing

**FIGURE 7 |:**
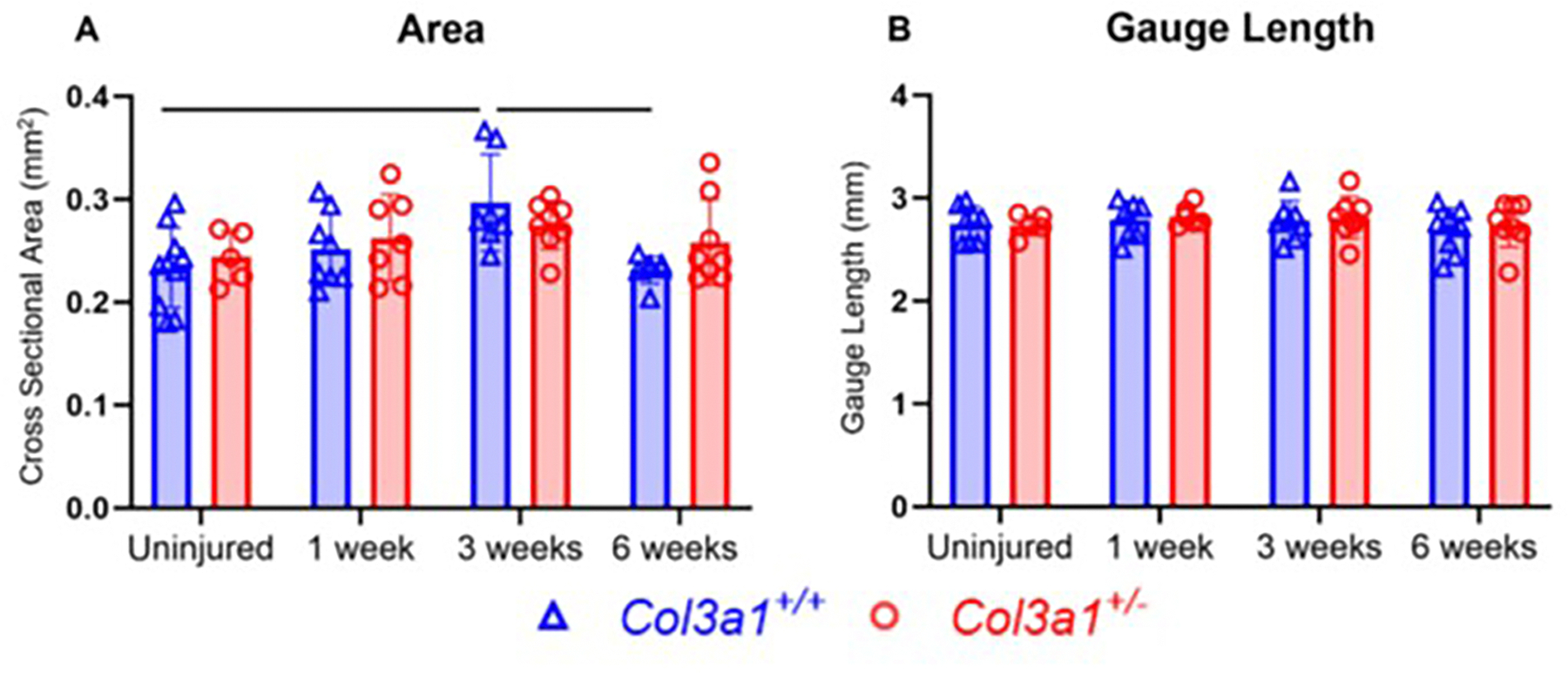
Tendon cross-sectional area dynamically responds to injury in *Col3a1*^+/+^ but not *Col3a1*^+/−^ mice, while gauge length remains unchanged by loss of COL3. (A) Cross-sectional area increases in *Col3a1*^+/+^ but not *Col3a1*^+/−^ tendons post-injury. (B) Gauge length did not change post-injury in either genotype. Solid line denotes significance of *p* ≤ 0.05. *n* = 7–10 tendons/genotype/time point.

Tendon cross-sectional area and length did not differ between genotypes (Figure 7A). Following injury, changes in area were not seen in COL3-deficient tendons, however, *Col3a1*^+/+^ tendon area was increased compared to uninjured tendon by 3-weeks post-injury (Figure 7A) with a return to uninjured area by 6-weeks post-injury. Loss of COL3 did not affect gauge length at any time point (Figure 7B).

**FIGURE 8 |:**
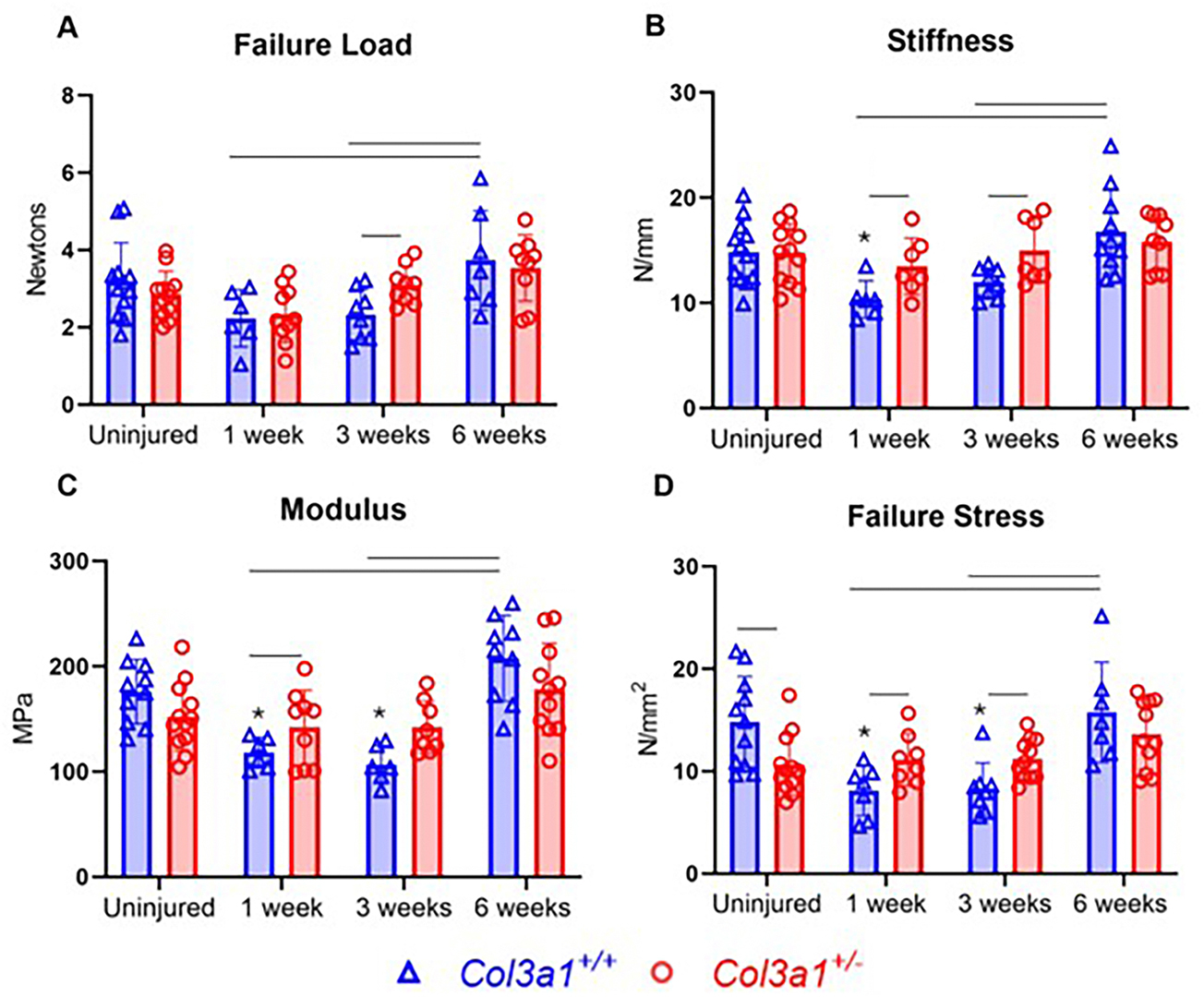
Tendon material and structural properties differ between uninjured and injured *Col3a1*^+/+^ and *Col3a1*^+/−^ tendons. (A) Failure load was increased at 3-weeks post-injury in *Col3a1*^+/−^ tendons. (B–D) Stiffness, stress and modulus were increased post-injury in *Col3a1*^+/−^ tendons at both 1- and 3-weeks post-injury. (A–D) Collectively, *Col3a1*^+/+^ properties decreased post-injury but returned to uninjured levels by 6-weeks, while *Col3a1*^+/−^ deficient tendon mechanical properties were unaffected by injury. Solid line denotes significance of *p* ≤ 0.05. *denotes significant difference between injured group and corresponding uninjured group. *n* = 7–10 tendons/genotype/time point.

COL3 loss did not affect failure load, stiffness, or modulus in uninjured tendons but resulted in a decreased failure stress in *Col3a1*^+/−^ tendons compared to *Col3a1*^+/+^ tendons (Figure 8A–D). Following injury, *Col3a1*^+/+^ tendon stiffness, modulus, and stress decreased by 1-week post-injury (Figure 8A–D, denoted by *), with the directional changes shifting by 6-weeks. At that time, increases in failure load, stiffness, failure stress and modulus were observed compared to both 1- and 3-week post-injury (Figure 8B–D). The comparable mechanical properties in uninjured tendons between genotypes further highlight differences post-injury and strongly implicate a role of COL3 in these mechanical property changes. Additionally, while failure stress is decreased in *Col3a1*^+/−^ uninjured tendons compared to *Col3a1*^+/+^, increased failure stress is seen post-injury, indicating a greater compensation. Interestingly, quasistatic properties in *Col3a1*^+/−^ tendons did not change at any time point post-injury from pre-injury levels (Figure 8B–D), resulting in genotypic differences during healing, including an increase in failure load at 3-weeks post-injury, an increase in stiffness and failure stress at 1- and 3-weeks, and an increase in modulus at 1-week post-injury in *Col3a1*^+/−^ tendons relative to *Col3a1*^+/+^ tendons (Figure 8B–D).

**FIGURE 9 |:**
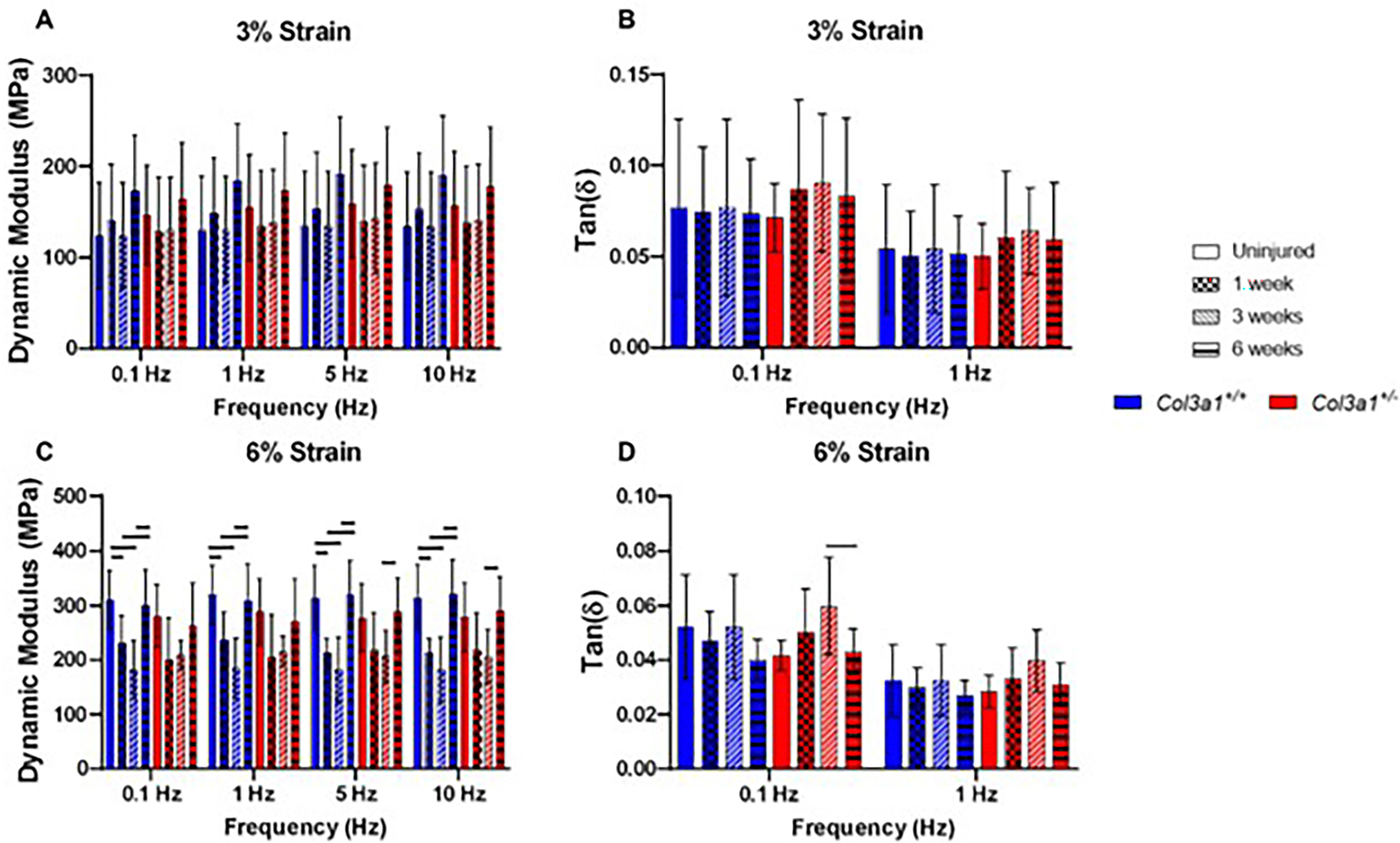
Col3 loss does not impact dynamic modulus and tan delta. (A,B) Tendons did not differ between genotypes or across injury time points in viscoelastic properties at 3% strain. (C) *Col3a1*^+/+^ tendons had reduced dynamic modulus values at 6% strain at 1- and 3-weeks post-injury. (D) No alterations in tanδ were seen, except between 3- and 6-weeks post-injury at 0.1 Hz in *Col3a1*^+/−^ tendons. Solid lines denote significance of *p* ≤ 0.05. *n* = 7–10 tendons/genotype/time point.

Tendons did not differ between genotypes or across injury time points in viscoelastic properties at 3% strain, however, changes can be seen at 6% strain (Figure 9A–D; Supplemental Figure 2). Percent relaxation did not differ between genotypes in uninjured or injured tendons (Supplemental Figure 2). Overall, there were no differences between genotypes post-injury, except a decrease from 3- to 6-weeks in percent relaxation of *Col3a1*^+/+^ tendons at 6% strain (Supplemental Figure 2).

**FIGURE 10 |:**
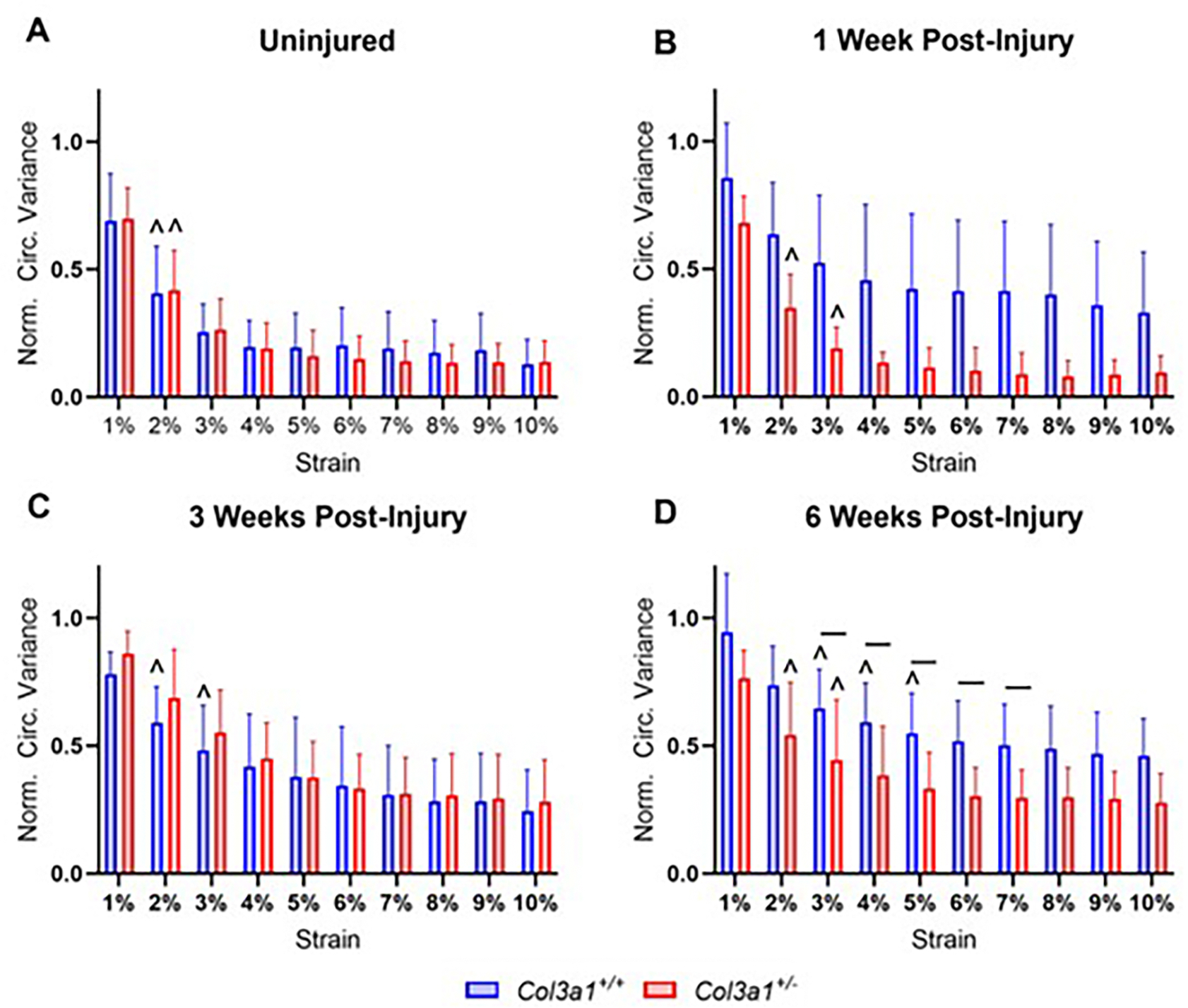
Collagen Fiber Realignment is impacted by COL3-deficiency post-injury. (A–D) Circular variance was measured to assess distribution of fiber alignment. COL3-deficient tendons realigned faster than *Col3a1*^+/+^ tendons at (B) 1- and (D) 6-weeks post-injury, and to a greater extent at 6-weeks post-injury. Solid line denotes significance of *p* ≤ 0.05. ^ denotes significant difference from the prior strain level. *n* = 7–10 tendons/genotype/time point.

Although there were no differences in realignment in uninjured tendons in response to load between genotypes (Figure 10A), COL3-deficient tendons realigned faster than *Col3a1*^+/+^ tendons at 1- and 6-weeks post-injury, and to a greater extent at 6-weeks post-injury (Figure 10B,D; realignment from prior strain level denoted by ^). At 3-weeks post-injury, *Col3a1*^+/+^ tendons realigned between 1%–2% and 2%–3% strain, while realignment did not change between any two subsequent strain levels in *Col3a1*^+/−^ tendons (Figure 10C).

### 3.8 | COL3-Deficient Tendons Exhibit Increased Fibril Sliding at 1% Strain in Uninjured Tendons

**FIGURE 11 |:**
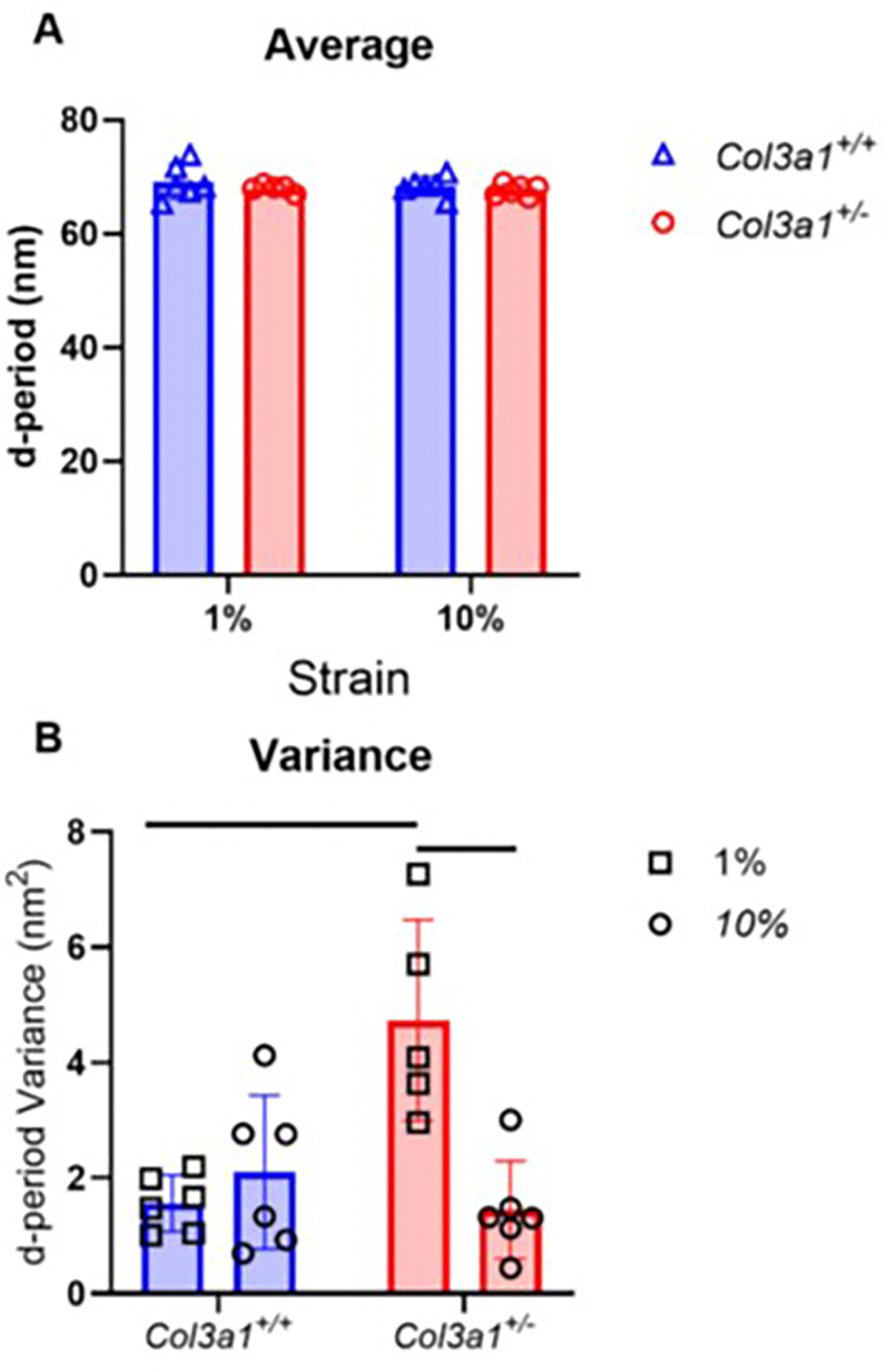
D-period variance, but not average is impacted by COL3 deficiency. (A) No difference in d-period average was seen between genotypes or across strain. (B) Variance was increased in COL3-deficient tendons at 1% strain compared to COL3-deficient tendons at 10% strain and *Col3a1*^+/+^ tendons at 1% strain. Statistical significance of *p* ≤ 0.05 is denoted by solid lines. *n* = 5–6 tendons/group.

D-period averages were similar in both genotypes at both 1% and 10% strain (Figure 11A). Variance of the -period in *Col3a1*^+/+^ tendons was not impacted by increased strain, however, COL3 loss increased d-period variance when tendons were loaded to 1% strain (Figure 11B). Additionally, when loaded to 1% strain, *Col3a1*^+/−^ tendons exhibited more sliding than when loaded to 10% strain, as indicated by increased dd-period variance (Figure 11B).

## 4 | Discussion

While the collagen composition of healthy tendon is primarily COL1, COL3 is induced with injury and becomes a major component of the healing provisional matrix [29]. Using a COL3 haploinsufficient mouse, we evaluated how COL3 loss impacts injured tendon matrix structure, composition, and organization and affects tendon mechanical and structural properties.

Our data suggests that COL3 likely has the ability to recruit and direct reparative cell activities and fate early in tendon healing, before shifting to a scar promoting role in the later stages of healing. While a robust but transient inflammatory healing phase has been associated with optimal healing, our data suggests loss of COL3 dampened the pro-inflammatory environment at 3-weeks post-injury. Notably, COL3 deficiency is associated with diminished *Scx* expression at 3-weeks post-injury, suggesting diminished tendon progenitors.

Consistent with our previous studies in cutaneous wounds and breast cancer [4, 9, 11], COL3-deficiency in the post-injury tendon microenvironment likely contributes to an increase in myofibroblast density. Remodeling by these myofibroblasts may be responsible for the observed more highly aligned matrix in healing *Col3a1*^+/−^ relative to *Col3a1*^+/+^ tendons. Notably, our expression data reveal that COL3 may play an important role in regulating other activated fibroblasts by suppressing fibroblast activation protein (*Fap*) at 1-week post-injury.

In addition to cellular activity, we investigated the impact of COL3 loss on the structure and function of ECM in uninjured and injured tendons. Interestingly, although there were no differences in *Col3a1* expression and COL3 content between genotypes in uninjured tendons at 90 days of age, a fibril diameter increase in tendons of *Col3a1*^+/−^ mice relative to *Col3a1*^+/+^ mice was observed and is consistent with the described role of COL3 in limiting fibril diameter in other tissues [3]. Differences in COL3 content may be present at earlier developmental points not examined, and could result in long-term effects on fiber structure [30]. Increased failure stress in *Col3a1*^+/+^ compared to *Col3a1*^+/−^ uninjured tendons is supportive of this concept. Additionally, high *Col3a1* expression early in tendon development indicates a potential role in initial fibril assembly [24]. Other studies have shown that ECM architecture, organization, and stiffness regulate cellular proliferation and migration indicating that a COL3-rich matrix may influence tendon development and healing [12, 31], emphasizing physical and mechanical characteristics, not solely organization. Therefore, the abundance of COL3 in the matrix may ultimately affect structural and functional properties. Further, despite higher alignment in *Col3a1*^+/−^ relative to *Col3a1*^+/+^ tendons at 6-weeks, mechanical properties are similar to uninjured levels, with no genotypic differences. Lack of genotypic differences may indicate that organization cannot compensate for alterations in composition and structure in terms of mechanical properties. Although mechanical properties recovered to uninjured levels by 6-weeks post-injury in both genotypes, alignment of the matrix did not approach uninjured levels by 6-weeks, likely indicating persistent scar. In addition to alignment [19, 32], altered fibrillogenesis [15, 30, 33, 34] likely contributes to increased mechanical properties in COL3-deficient tendons. Although studies in meniscus and cartilage [5] have shown disruption of the matrix structure outweighs the fibril stiffening impacts, we observe the opposite, highlighting the multifaceted role of COL3 and its unique contributions to various soft tissues.

To examine matrix engagement, collagen realignment was evaluated. *Col3a1*^+/−^ tendons realigned faster at 1- and 6-weeks post-injury, and to a greater degree at 6-weeks post-injury than *Col3a1*^+/+^ tendons. While material and structural properties did not differ 6-weeks post-injury, collagen realignment is faster in *Col3a1*^+/−^ compared to *Col3a1*^+/+^ tendons at this time point. Alignment allows for faster fiber engagement when loaded [19]. At a micro-level, faster fiber realignment under loading conditions likely correlates with increased inter-fibrillar interactions and matrix engagement [20]. Although healing tendons in *Col3a1*^+/−^ mice relative to *Col3a1*^+/+^ littermates have reduced COL3 content at 1- and 3-weeks post-injury, no differences were noted at 6-weeks. Similar to responses in other tissues [11], increased myofibroblast density associated with COL3-deficiency at 3-weeks post-injury may lead to an increase in COL3 expression to obscure differences at 6-weeks in this model, as well as explain the increased stiffness and contraction of the matrix in *Col3a1*^+/−^ relative to *Col3a1*^+/+^ tendons post-injury.

Additionally, stiffer fibers realign faster [35] further supporting increases in quasistatic mechanical properties. At 3-weeks post-injury, while *Col3a1*^+/−^ tendons are stiffer, with higher failure loads, realignment with load is more gradual. In the toe region, *Col3a1*^+/+^ tendons realign faster, with similar rates between genotypes once in the linear region. Matrix organization plays a crucial role in viscoelastic properties [36].

We hypothesize that a more highly aligned matrix overall preserves the quasistatic mechanical properties [37], explaining why COL3-deficient tendons were unaffected by injury in these parameters. Early in healing, enhanced alignment in COL3-deficient tendons may promote realignment under load and result in increased modulus, stress and stiffness in these tendons compared to *Col3a1*^+/+^.

Three weeks post-injury, the remodeling phase becomes superimposed on the active but resolving proliferative phase [38], making this a complex time point. ECM architecture plays a key role in matrix behavior, strain transmission and load response [39]. COL3-deficiency resulted in increased collagen fibril diameters in uninjured tendons, supporting our original hypothesis. A slightly increased population of larger fibrils at 3-weeks post-injury in *Col3a1*^+/−^ tendons likely influenced mechanical property increases when compared to *Col3a1*^+/+^ tendons, supported by previous literature showing that larger fibril diameters can withstand greater loads and compensate for injury [40]. Increasing stiffness with increasing fibril diameter is supported, as increased fibril diameters increase tendon macroscale stiffness [40]. Additionally, increased myofibroblast activation and realignment has been implicated in increased fibril-fibril engagement and efficiency of load transfer [41]. Alignment is not different at this time point between genotypes, which may indicate that increased engagement and architecture influences the mechanical properties more than organization. Interestingly, by 6-weeks post-injury, the *Col3a1*^+/−^ tendon fibril diameter population remains similar to 3-weeks, while the 6-week *Col3a1*^+/+^ fibril diameter population shifts to larger fibrils compared to the 3-week *Col3a1*^+/+^ fibril population. This supports literature showing that *Col3a1*^+/+^ fibrils continue fibrillogenesis later post-injury, while *Col3a1*^+/−^ fibrils lateralize earlier in the healing process due to dysregulation of fibrillogenesis from COL3-deficiency [5]. Fibrillogenesis begins with assembly of intermediate molecules, progresses with nucleation, or beginning of growth, and finishes with longitudinal and lateral growth [6, 42–44]. By 6-weeks, *Col3a1*^+/+^ tendons surpass *Col3a1*^+/−^ tendons in fibril diameter growth, and this may explain why genotypic differences in mechanics are no longer seen at that time.

Analysis of *Col3a1*^+/+^ compared to *Col3a1*^+/−^ tendons across strain levels via AFM revealed an interaction between genotype and strain level, indicating that strain is affecting genotypes differently. If the fibrils are more highly aligned, less activation energy is needed to engage the fibrils, and sliding will occur [45]. Increased fibril sliding occurs in *Col3a1*^+/−^ tendons, which supports faster realignment in *Col3a1*^+/−^ tendons during the mechanical testing protocol.

While comprehensive, this study is not without limitations. Although we evaluated an extensive list of genes, and examined appropriate cellular markers via immunohistochemistry, the sample sizes used for the Fluidigm assay and immunofluorescence, may limit the ability to distinguish all biologically relevant differences. Future work may include further examination of gene expression with increased time points post-injury. In particular, it is accepted that IL-33 likely plays a role in fibrosis during tissue remodeling [44] and recent literature points to IL-6 influencing chronic tendon disease and fibrosis through fibroblast signalling [45]. Our data supports that COL3 deficiency leads to an induction of *IL-6* at 1-week post-injury and a decrease in *Il-33* expression at 3-weeks, however it is possible that an earlier time point might reveal an active IL-33/IL-6/COL3 axis [46–48] following tendon injury. Lastly, COL3-deficiency appears transient in tendons in this heterozygous *Col3a1* knockdown model. This model was used due to the perinatal lethality of homozygous *Col3a1*^−/−^ mice and, at the time of the study, the lack of a conditional *Col3a1* knockout model. Current studies utilizing a newly created *Col3a1*^*F/F*^ model will build on this foundational work and allow investigation of the effect of COL3 knockdown on tendon repair with temporal regulation [4]. Overall, our data suggests that COL3 plays a pro-healing role early in tendon repair but a scar-promoting role in late tendon repair. Future studies will be important to more clearly determine the impact of COL3 on individual phases of healing and spatial restriction of COL3 knockdown.

## Supplementary Material

Suppl Figure 2 **Figure S2:** Stress Relaxation does not differ between uninjured and injured tendons of WT and *Col3a1*^+/−^ mice.

Suppl Figure 1 **Figure S1:** COL3-deficiency does not impact collagen fiber straightness, length or width in post-injury tendons.

Supporting Information

Additional supporting information can be found online in the Supporting Information section.

## Figures and Tables

**TABLE 1 |:** Gene panel for 96.96 microfluidic qPCR array.

Collagens	Non-collagenous matrix	Matrix (Re) modeling & modification	Cell-ECM interactions	Signaling pathways	Cell markers	Inflammation	Cell cycle	Housekeeping
Col1a1, Col1a2, Col2a1, Col3a1, Col5a1, Col6a1, Col6a2, Col11a1, Col12a1, Col14a1	Acan, Aspn, Bgn, Dcn, Fap, Fbn1, Fmod, Fn1, Hspg2, Kera, Lum, Postn, Prg4, Sparc, Thbs2, Thbs4, Tnc, Tnmd, Vcan	Adamts1, Adamts15, Loxl1, Loxl2, Mmp2, Mmp3, Mmp7, Mmp8, Mmp9, Mmp13, Mmp14, Timp1, Timp3	Cdh11, Gja1, Itgb1, Itga1, Itga2, Itga5, Itgb5, Itga11, Itgv, Plod2, Plod3	Bmp4, Ctgf, Egr1, Ltbp1, Ltbp4, Mstn, Taz, Tgfb1, Tgfb2, Tgfb3, Tgfbr2, Yap1	Acta2, Adgre1, Cd3e, Cd31, Cd38, Cd68, Cd163, Mkx, Mpo, Scx, Sox9, Vim	IL1B, IL6, IL10, IL12, IL13, IL33, TLR2, TLR4, TNFa	Axin2, Casp3, H2AFX, Pcna	18S, Abl1, Rps17

## Data Availability

The data that support the findings of this study are available from the corresponding author upon reasonable request.
